# Painfully thin but locked inside a fatter body: abnormalities in both anticipation and execution of action in anorexia nervosa

**DOI:** 10.1186/1756-0500-7-707

**Published:** 2014-10-09

**Authors:** Morgane Metral, Dewi Guardia, Ines Bauwens, Michel Guerraz, Gilles Lafargue, Olivier Cottencin, Marion Luyat

**Affiliations:** LPNC (Laboratory of Psychology and Neurocognition), UMR5105, F-73000 Chambéry, France; LNFP (Laboratory of Functional Neuroscience and Pathology) EA4559, F-59000 Lille, France; Addiction Center, CHRU Fontan2 (Regional University Hospital), F-59000 Lille, France; Department of Psychology, University of Lille 3, F-59650 Villeneuve d’Ascq, France; Adolescent Mental Health Center, Lautreamont Clinic, F-59120 Loos, France

**Keywords:** Anorexia nervosa, Body representation, Motor imagery, Body schema, Body image, Body-scaled action

## Abstract

**Background:**

People with anorexia nervosa (AN) usually report feeling broader than they really are. The objective of the present study was to better understand the body schema's involvement in this false self-representation in AN. We tested the potential for correction of the body schema impairment via the sensorimotor feedback provided by a real, executed action and relative to an imagined action. We also took account of the impact of the AN patients’ weight variations on the task outcomes.

**Methods:**

Fourteen inpatient participants with AN and fourteen control participants were presented with a doorway-like aperture. The participants had to (i) judge whether or not various apertures were wide enough for them to pass through in a motor imagery task and then (ii) actually perform the action by passing through various apertures.

**Results:**

We observed a higher passability ratio (i.e. the ratio between the critical aperture size and shoulder width) in participants with AN (relative to controls) for both motor imagery and real action. Moreover, the magnitude of the passability ratio was positively correlated with weight recovery.

**Conclusion:**

The body schema alteration in AN appears to be strong enough to affect the patient's actions. Furthermore, the alteration resists correction by the sensorimotor feedback generated during action. This bias is linked to weight variations. The central nervous system might be locked to a false representation of the body that cannot be updated. Moreover, these results prompt us to suggest that emotional burden during weight recovery could also alter sensorimotor aspects of body representation. New therapeutic methods should take account of body schema alterations in AN as adjuncts to psychotherapy.

## Background

"Being so many different sizes in a day is very confusing". Just as with Alice in Wonderland, body mass changes may not only affect a person's feelings about his/her body but may also affect the sensorimotor system. Both before and after massive, rapid weight loss (as in anorexia nervosa (AN), a serious mental illness that predominantly affects young women [1]), patients often consider that they are fatter and broader than they really are [2]. This *"disturbance in the way in which one's body weight or shape is experienced"* is a major clinical symptom of AN [3] but is also a risk factor that drives the obsessive will to lose weight and contributes to the maintenance of restrictive eating behaviors [4]. Thus, there is an urgent need to gain a better understanding of the process underlying body overestimation in AN, with a view to developing novel remediation techniques as adjuncts to psychotherapy.

From a psychopathological point of view, factors such as attachment style (insecurity of attachment and the need for approval) and depressive symptoms (negative judgments about oneself, worshipping others) have a crucial role in body dissatisfaction [5] and are probably also linked to body overestimation [6]. From a neuropsychological point of view, body overestimation in AN provides clues as to how the body representation is built and then adjusted as a function of weight changes and related to body width changes [7]. In this context, researchers draw a distinction between two major body representations (for a review, see [8]): the body image and the body schema. Body image is considered to be a conscious representation of the body in terms of its esthetical, emotional and social components. In contrast, body schema is viewed as a sensorimotor representation of the body that is involved whenever a motor action is imagined or performed [8–11]. Many of the studies to date have stressed the disruption of the conscious body image in AN [12]. Only a few authors have suggested that the involvement of a body schema distortion [7, 13–17] is related to the putative dysfunction of the parietal cortex (and the right parietal lobule in particular) in AN [18, 19], since that this cortical area was found to be crucial for emergence of the neural network that establishes a coherent body schema [20].

Considering that body schema is involved in the overall process of action (from the anticipation through to execution) and in view of literature evidence that simulation of action by motor imagery and motor execution share kinematic and neural properties [21–23], our group first looked at possible repercussions of this abnormal body schema in AN by using a first-person visuomotor imagery task [15, 16]. In fact, we applied an aperture passability task similar to that used in Experiment 2 of a seminal study [24]. This paradigm has been further used to investigate emotional/social influences on spatial perception [25] and to evidence motor dysfunctions in Parkinson's disease [26, 27]. In our previous experiments [15, 16], AN patients and control participants had to judge (without executing the action) whether or not an aperture was wide enough for them to pass through. Hence, the patients judged that they could not pass through an aperture without turning their shoulders, even when it was easily wide enough for them. In fact the patients responded as if their body was larger than it really was - confirming the clinical complaint of feeling fat that has been evaluated with various body image questionnaires [28–30] and contour drawing scales [31, 32].

Although these motor imagery abnormalities were relevant for highlighting the involvement of the body schema, the importance of testing the impact of these abnormalities on real actions was suggested by Guardia et al. [16] and tested in a case study [7]. Indeed, Keizer et al. [17] recently investigated whether disturbed experience of body image also extended to body schema in a body-scaled action in AN. To better understand body schema changes as a core symptom of AN, we decided to further investigate the role of action in this disorder by comparing imagined and real action in a same experiment. Indeed, humans demonstrate a remarkable ability to generate accurate, appropriate motor behavior under the changing environmental conditions that they encounter in daily life. It has been suggested that the central nervous system uses a modular approach in which multiple controllers co-exist and are selected according to the context of the movement (such as the size of surrounding objects that could be obstacles [33]). According to this hypothesis and in line with Frith’s model [34], the desired action (passing through an aperture, for instance) creates a motor sequence that can be adjusted during the realization of action by the motor control system as a function of the environmental constraints (the size of an aperture, for instance). People with AN may misperceived the critical aperture when simulating the action (i.e. in a motor imagery task) because their internal model is based on an incorrect body schema. However, if they pass through many different apertures in a given session, AN patients might be able to correct their pattern of movements and compensate for their impaired body schema in a "learning-by doing" process [35]. Moreover, testing the execution of an action is an implicit means of evoking the body schema by preventing top-down processes (such as the intrusion of an impaired conscious body image). This approach is especially pertinent in AN, in view of the recent suggestion [36] that patients are locked to a predominant, allocentric body image related to their previous body size and weight (possibly due to rapid and massive weight loss). Hence, this allocentric body image might alter the updating of their new body schema.

Thus, in the present study, we sought to test the hypothesis whereby AN patients not only *think* they are fatter but *behave* as if they were fatter in an activity of daily life because they are unable to correct their internal model via external and proprioceptive inputs provided by execution of the action. Hence, we sought to establish whether anorexic patients not only *misperceive* the critical aperture but also *behave* wrongly accordingly. To this end, we recorded the actual gait and movements of AN patients as they approached and passed through different apertures (i.e. the real action). A study of a real action task [24] showed that there is a critical aperture width through which the healthy participant could no longer pass without turning sideways to some extent (a πi ratio of 1.3). This value means that the participant first turns sideways when the aperture is 1.3 times wider than the participant's shoulder width. Moreover, this rotation is initiated before the passage and is body-scaled. These actions were also compared with the results of the mental imagery task. Moreover, our method is quantitative and enables a statistical analysis of the clinician's observations concerning body overestimation and dissatisfaction. Our starting hypothesis was that if body schema is affected in AN, patients not only overestimate their critical aperture more than control participants do but also behave as if their bodies were wider by turning their shoulders for a wider critical aperture than control participants do. In both imagery and action, the passability ratio should be higher in the AN group than in the control group.

## Methods

### Ethics statement

This study was approved by a regional independent ethics committee (Comité de Protection des Personnes Nord-Ouest IV; study number: 2007-A01413-50). The study complied with the tenets of the Declaration of Helsinki. Each participant received a study information sheet and provided her written, informed consent to participation. Parental consent was additionally required for participants under the age of 18.

### Participants

The demographic and clinical characteristics of the study population are summarized in Table 1. The study included 28 young female participants: 14 AN inpatients recruited at the hospital and 14 healthy, controls (matched for educational level and age), recruited from the hospital's medical students and medical staff. Neither AN patients nor controls had participated in our previous experiments. Although the study participants had been informed about the study's general objective and procedures, they were not told about the starting hypotheses or the expected results. All 14 patients fulfilled the DSM IV-TR criteria for AN, with 12 restricting types [37]. Administration of the Mini-International Neuropsychiatric Interview [38] by a psychiatrist confirmed the absence of other psychiatric pathologies (according to the DSM IV criteria) in the two groups. All the control participants had a normal BMI (i.e. weight/height^2^ ranging from 18.5 to 25 kg/m^2^). Male AN sufferers were not recruited, given their low prevalence and the high rate of psychiatric comorbidities in this population. A neuropsychiatric interview of the participants did not reveal any perceptual, attentional or intellectual impairments. The participants were asked to mention all ongoing courses of medication or other treatments (such as physiotherapy, osteopathy, and psychomotricity) and any previous hospitalizations due to sensorimotor problems. People with a history of neurological, ophthalmic or sensorimotor problems or with ongoing or previous treatments with strongly psychotropic medications were excluded. The experiment lasted approximately one and a half hours.

**Table 1:** **Demographic and clinical data for the anorexia nervosa (AN) and control groups**

	AN group			Control group			
	Mean	SD	Median	Mean	SD	Median	P
Age (years)	24.14	9.65	22	25.21	7.77	24	0.74^a^
Height (m)	1.66	0.05	1.67	1.67	0.08	1.68	0.85^a^
Shoulder width (cm)	36.07	1.54	35.7	40.66	3.23	39.75	<0.001^b^
Weight (kg)							
*Current*	40.42	3.78	39.85	60.04	.74	59	<0.0001^b^
*Minimum reached (n*_*AN*_ *= 13)*	36.98	4.31	37	-	-	-	-
*Pre-disease(n*_*AN*_ *= 14)*	56.50	5.13	56	-	-	-	-
*1 month before (n*_*AN*_ *= 13)*	39.28	3.42	39.1	59.64	6.62	59	<0.0001^b^
*6 month before (n*_*AN*_ *= 13)*	41.72	7.59	40.5	59.61	7.25	59	<0.0001^b^
BMI (kg/cm^2^)							
*Current*	14.70	1.50	14.76	21.62	1.82	21.78	<0.0001 ^a^
Minimum reached (nAN = 13)*	13.26	1.30	13.31	-	-	-	-
*Difference (nAN = 13)*	1.28	1.10	1.26	-	-	-	-
BSQ score	126.93	42.36	137.5	80.50	24.63	80.5	0.002^b^
EDI-2							
*Total score*	110.79	34.23	112.5	34.93	16.87	34.5	<0.0001^b^
*DT subscale score*	12.21	7.18	14.5	3.21	4.46	2	<0.001^b^
*BD subscale score*	17.71	7.32	19	7.14	5.86	6.5	<0.001^b^
Disease							
*Years since onset*	5.71	9.67	2	-	-		-

BMI: body mass index; BSQ: Body Shape Questionnaire; EDI-2: Eating Disorder Inventory, second version; DT: drive for thinness; BD: body dissatisfaction; (^a^): T-test; (^b^): Mann–Whitney U-test. *n = 13 for the data concerning the weights because, for one participant, the medical staff could not find the different weights due to the patient’s hospitalization in different institutions.

### Materials and procedures

#### Morphological and clinical parameters

An investigator measured each participant's height and shoulder width. The "bi-acromial" shoulder width was measured with a medical measuring rod with 1 mm graduations, and corresponded to the distance between the outside edges of the shoulders' acromion processes. In view of the patients' emotional burden, bodyweight was measured twice a week by hospital staff (as part of the patient's normal care) and not by the investigators. The last body weight indicated in the patient's medical records was considered to be the current value. Changes over time in nutritional states were measured by considering the patient's current body weight and the values measured by medical staff one month and six months before the study (which were also available in the patients’ medical records). Only the weight just before the beginning of the dieting and weight loss period (i.e. just prior to the onset of AN) was self-reported. Body dissatisfaction and concerns about weight and shape were assessed in both control and AN groups by administering the Body Shape Questionnaire (BSQ) and the Eating Disorder Inventory-2 (EDI-2), respectively. On the day of the study, these questionnaires were administered at the end of the experiment (to avoid emotional arousal and perturbation during experimental tasks).

The BSQ [29] is an one-dimensional, 34-item self-questionnaire that assesses concerns about body shape over the preceding 4 weeks. Answers are scored on a 6-point Likert scale of 1 (not present) to 6 (always present). The EDI-2 [30] is a self-questionnaire with 11 subscales measuring psychological features commonly associated with eating disorders. Ninety-one items are rated on a Likert scale of 1 (never) to 6 (always).

#### Evaluation of passability

The "doorway-like" apertures were formed by two vertical poles (height: 1.80 m). The participant first stood 5 m away the aperture. The width between the two poles could be adjusted to between 36 cm to 80 cm in stepwise, 2 cm increments (in increasing and decreasing series). Two experimental conditions were tested.

In the *motor imagery condition*, the participants were instructed to imagine (without moving from their starting position) whether or not they would be able to walk through the presented apertures. The participants were told to answer *"*yes*"* if they thought it would be possible for them to pass through the aperture at a normal walking speed (as if they were at home, walking from one room to another) without having to turn sideways or to change their posture. If they thought that this was not the case, the participants were told to answer *"no"*. The imagery was repeated twice with increasingly narrow apertures (yielding two descending series) and twice with increasingly wide apertures (yielding two ascending series). A descending series was stopped when there was a transition from a "yes" reply to two consecutive "no" replies. An ascending series was stopped when there was a transition from a "no" reply to two consecutive "yes" replies.

**Figure 1:**
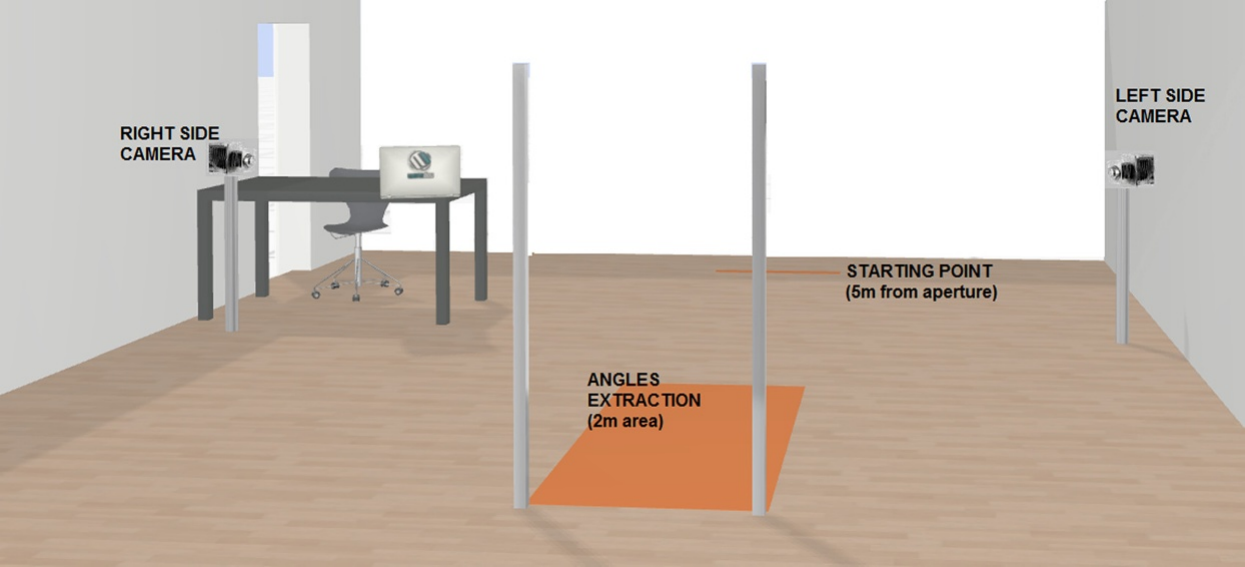
**The experimental set-up during the action task.**

In the *action condition*, participants were instructed to walk through the aperture at a comfortable pace and to turn sideways if they so wished (in line with [24]). Next, they were asked to go as far as the wall behind the aperture, stop for 5 seconds, turn around and returned to the starting position by passing through the aperture a second time. In both groups, action ratios were always greater than 1 (the lowest was 1.04), meaning that none of the participants touched the poles. The condition was performed once with increasingly narrow apertures (yielding a descending series) and once with increasingly wide apertures (yielding an ascending series). The participant was filmed with two cameras (50 Hz) - one on the left side of the room in which the experiment was performed, and the other on the right side. These provided objective measures of shoulder rotation for each passage through the aperture. The starting point (5 m from the aperture) was the same for all participants and was marked on the ground. It was parallel to the aperture and centered relative to the left and right poles, so that the participant's approach was perpendicular to the aperture. For each aperture, we looked for shoulder rotation from about 2 m from the aperture; 2 m is the distance in which abnormally high shoulder rotation (i.e. greater than natural body sway) usually appears when approaching a critical aperture or a narrower aperture [39]. The room's lay-out is shown diagrammatically in Figure 1.

The motor imagery condition was always performed before the action condition. The action condition could not be performed first, since it would have improved performance in the motor imagery condition. In both conditions, participants had to close their eyes between trials so that they could not (i) see the poles being adjusted or (ii) using the experimenter’s body as a benchmark for judging the width of the aperture. Participants did not receive feedback on their performance.

#### Measurements of critical apertures and ratios

For both motor imagery and action conditions, we calculated a passability threshold (i.e. the critical aperture) and a ratio.

The *motor imagery* threshold (i.e. the critical aperture in motor imagery condition) in each series was defined as the average width at which the participants thought it was no longer possible to pass through without turning the shoulders. For each series, the critical aperture was the mean of the two successive aperture widths for which a change in reply ("yes, I could pass through the aperture without turning my shoulders" or "no, I could not") was obtained. We then computed the mean motor imagery critical aperture over the four motor imagery series. Lastly, a motor imagery ratio (πi) was computed by dividing the average motor imagery critical aperture by the participant’s shoulder width.

**Figure 2:**
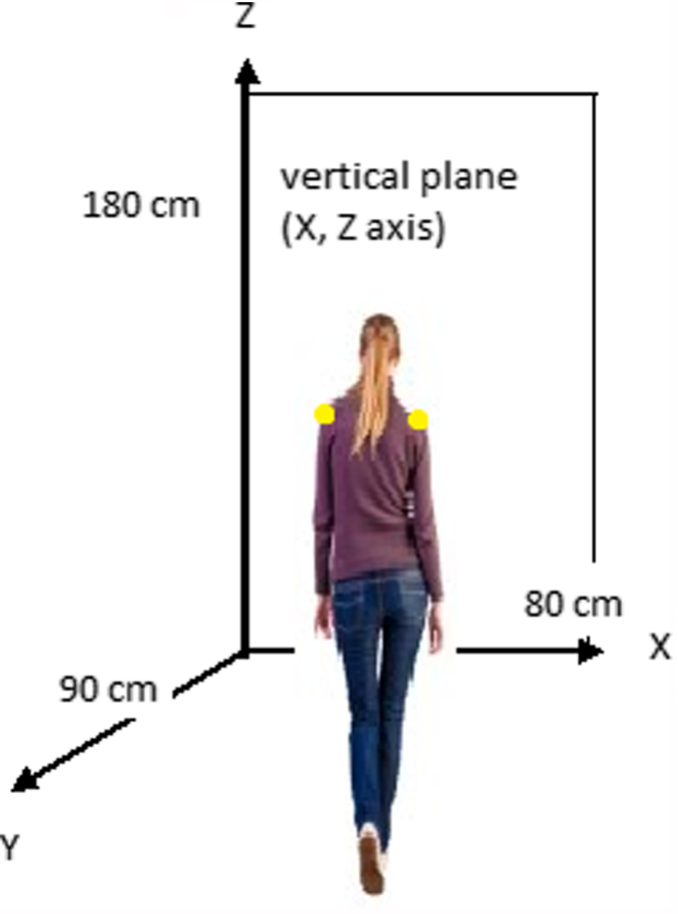
**Diagram of the calibration space and the vertical plane used for angle measurement (as viewed by the participant).**

The *motor* threshold (i.e. the critical aperture in the action condition) was the mean width at which the subject began to turn her shoulders to an extent that exceeded a baseline level of natural rotational body sway [39]. The two cameras used the same time code and were synchronized electronically via a control box (model DT9817, Data Translation, Marlboro, MA, USA). Video data were processed with three-dimensional motion capture and analysis software (Simi Motion®, Simi Reality Motion Systems GmbH, Unterschleissheim, Germany). We used a cubic, calibrated space measuring 90 cm in length (the y axis), 80 cm in width (the x axis) and 180 cm in height (the z axis) (see Figure 2). The cameras were calibrated before each participant's test session. The shoulder rotation angles were determined from a spherical light sensor positioned on each of the participant's shoulders. These angles were compared with the aperture's plane (i.e. the vertical plane defined by the x and z axes; see Figure 2).

**Figure 3:**
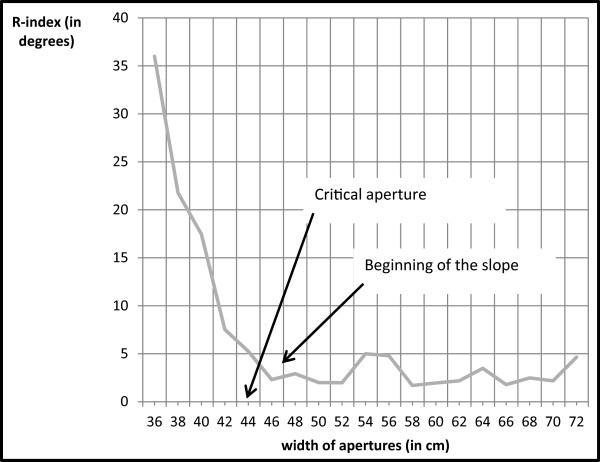
**The R-index (the maximum rotation angle minus the mean deviation of all the rotation angles) as a function of aperture width.**

The measurement accuracy for the shoulder movements was 0.5 cm. The angle was extracted for each captured image (yielding 25 angles per second, given that the cameras’ frame rate was 50 Hz). Shoulder rotation angles could be either positive or negative, due to the body's natural left and right rotational sway during locomotion. Given that we wanted to focus on the emergence of shoulder rotation, we were interested in absolute values and their variation. Hence, we tried to determine the magnitude of shoulder rotation as accurately as possible if it occurred. For that, we chose to consider deviations above a predefined baseline rotation, as per [39]. For each aperture width, we then calculated a shoulder rotation index (referred to as henceforth as the R-index) as follows: (maximum angle in the dataset) - (mean deviation). The *maximum angle* was the largest absolute angle (in degrees) recorded for a given passage and aperture width. The *mean deviation* was the mean deviation between the absolute values of the various angles measured during gait and the mean of these angles. After having computed the R-indexes for each ascending or descending set, the critical aperture was defined graphically (as per [24]) as the first aperture after the inflexion point in the curve (see Figure 3).

Two laboratory members (acting as independent observers) were blinded to the participant's status (AN or control) and determined the critical aperture, as defined above. The correlation coefficient *r* for inter-rater agreement was 0.98. We then computed the mean motor critical aperture over the two series. Lastly, an action ratio (πa) was computed by dividing the motor critical aperture by the participant’s shoulder width.

### Statistical analysis

All analyses were performed with Statistica software (version 7.1, Statsoft Inc., Tulsa, OK). When the conditions for application of a parametric test were fulfilled (i.e. a normal data distributions and homogeneity of variances), we applied Student's T-test or an analysis of variance (ANOVA) when comparing means and the Bravais-Pearson test when calculating correlations. Non-parametric Mann–Whitney and Spearman tests were used when non-normal distributions and/or non-homogenous inter-group variances were observed.

## Results

### Morphological and clinical parameters

Our analysis of the participants' morphological and clinical parameters is summarized in Table 1. There were no significant inter-group differences in terms of age (Mean_AN_ ± SD =24.14 ± 9.65 years *vs.* Mean_C_ ± SD = 25.21 ± 7.77 years, t_26_ = 0.32, p =0.74), or height (Mean_AN_ = 1.66 ± 0.05 cm *vs.* Mean_C_ = 1.67 ± 0.08 cm, t_26_ = 0.24, p = 0.85). In contrast, the mean current BMI was significantly lower for AN patients (Mean_AN_ = 14.70 ± 1.50) than for controls (Mean_C_ = 21.62 ± 1.82, t_26_ = 10.98, p <0.0001). Median shoulder width was also significantly lower in the AN group (Med_AN_ = 35.7 cm) than in the control group (Med_C_ = 39.75 cm, U =13.50, Z =3.88, p =0.001), reflecting the patients' state of malnutrition.

The mean time since disease onset was 5.71 ± 9.67 years. When compared with the control participants, the AN group had a significantly higher EDI-2 total score (Med_AN_ = 112.5, Med_C_ = 34.5; U =10, Z = -4.04, p <0.0001) and subscale scores ("drive for thinness"; Med_AN_ = 14.5; Med_C_ = 2, U =32.50, Z = -3.01, p = 0.002; "body dissatisfaction"; Med_AN_ = 19, Med_C_ = 6.5, U =26, Z = -3.30, p = <0.001). The BSQ scores were also significantly greater in the AN group than in the control group (Med_AN_ = 137.5, Med_C_ = 80.5, U =37.50, Z = -2.78, p =0.005). In order to analyze the effect of weight changes, two measurements were taken into account: the lowest BMI reached during the course of the disease (Mean_AN_ =13.26 ± 1.30) and the difference between the present BMI and the lowest BMI (Mean_AN_ =1.28 ± 1.10).

### Evaluation of passability

The results for the motor imagery and action tasks are summarized in Table 2.

**Table 2:** **Critical aperture, imagery and action ratios in the anorexia nervosa (AN) group and in the healthy control group**

	AN group				Control group				
	(n = 14)				(n = 14)				
	Mean	SD	Median	Min; Max	Mean	SD	Median	Min; Max	p
Critical aperture									
*Motor imagery task*	44.32	6.11	45.75	9.5 ; 58.5	46.64	4.94	42.00	6.0 ; 56.5	0.28(a)
*Action task*	7.35	3.24	48.00	3.0; 52.0	47.75	3.00	48.00	0.0 ; 53.0	0.74(a)
Ratio									
*Motor imagery task*	1.29	0.17	1.27	1.05 ; 1.62	1.09	0.10	1.09	0.90 ; 1.21	<.0001(b)
*Action task*	1.32	0.07	1.34	1.13 ; 1.41	1.16	0.07	1.16	1.05 ; 1.32	<.0001(b)
First set	1.29	0.08	1.30	1.13 ; 1.38	1.16	0.11	1.13	1.02 ; 1.42	
Second set	1.35	0.11	1.37	1.13 ; 1.51	1.18	0.06	1.17	1.06 ; 1.28	

Critical aperture in the motor imagery task: the mean width at which the participant thinks it is no longer possible to pass through without turning the shoulders. Critical aperture in the action task: the mean width at which the participant begins to turn their shoulders by more than a baseline level of natural body sway. Ratio in the motor imagery task: the motor imagery critical aperture divided by the shoulder width in the motor imagery condition. Ratio in the action task: the action critical aperture divided by shoulder width in the action condition. (a): in a T test; (b): in a Mann–Whitney U test.

In the imagery condition, the individual πi ratios were much higher in most patients than in controls, with median ratios of 1.27 and 1.09 for the respective groups (U = 26, Z = -3.30, p < 0.001). Similar differences were observed in the action task, with higher πa ratios in most patients than in controls. The median ratio was 1.34 in the AN group and 1.16 in the control group. The significant difference in the action ratio (π_a_) was confirmed by statistical analysis (U =19, Z = -3.63, p <0.001).

**Figure 4:**
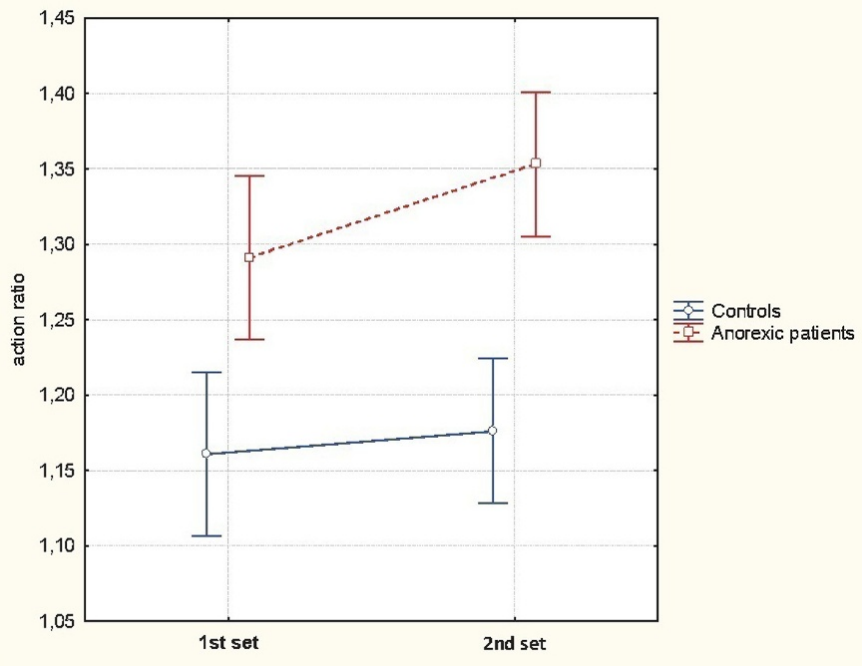
**The mean action ratio as a function of the series (i.e. the first series vs. the second series) and the group.** AN = patients suffered from anorexia nervosa. The vertical bars represent the 95% confidence interval.

In order to establish whether the AN group's ratio could be improved by repeating the action, we performed a 2×2 analysis of variance with repeated measures on the *series* factor and with *group* as a categorical predictor for the action ratio. This ANOVA revealed a significant effect of group (F_(1,26)_ = 27.76, p < 0.0001). The AN group had significant higher action ratio (Mean = 1.32) than the control group (Mean = 1.16). Moreover, the effect of *series* was not statistically significant (F_(1,26)_ = 3.80, p = 0.06). The first series yielded a lower action ratio (Mean = 1.23) than the second series (Mean = 1.26). The interaction between *series* and *group* was not statistically significant: F_(1,26)_ = 1.40, p = 0.25 (see Figure 4). However, an *a priori* comparison revealed that the lower action ratio in the AN group was significantly lower in the first series (Mean = 1.29) than in the second series (Mean = 1.35). In contrast, no such difference was observed for the control group when comparing the first series (Mean =1.16) with the second series (Mean = 1.18; F_(1,26)_ < 1).

Calculation of Spearman’s correlation coefficient (ρ) revealed a positive relationship between the motor imagery ratio and the action ratio for the study population as a whole (n = 28; ρ = 0.55; p < 0.05; one-tailed test). Our analysis did not reveal a significant correlation between the participant's height and the passability ratios for either the motor imagery condition (ρ = -0.21; p > 0.20) or the action condition (ρ = -0.17; p > 0.20).

In the AN group, the imagery ratio (πi) was not related to the BMI before the disease (ρ = 0.06; p >0.50), the BMI one month before the study (ρ = -0.01; p > 0.50) or the BMI weight six months before the study (ρ = -0.21; p > 0.20). However, πi was related to the difference between the BMI at the date of the experiment less the lowest BMI reached during the disease (weight recovery; ρ = 0.55, p <0.05 in a one-tailed test). Likewise, there were no significant correlations between these variables and the action ratio (πa) (all p > 0.10).

## Discussion

In terms of perception of one's own body, patients suffering from AN have a very high level of body dissatisfaction. This reflects many different psychological factors. Attachment style and depressive symptoms were found to be more predictive of body dissatisfaction than personality traits were [5]. Body overestimation (i.e. a persistent feeling of being fatter than in reality) is linked to body dissatisfaction [6] and is a very complex symptom. It reflects many different false representations of the body (i.e. body schema as well as body image). Conscious, emotional and esthetic body overestimation may be embedded in the body schema at the sensorimotor level. To investigate the body schema's role in AN, we used ecological tasks in which the participant had to judge her likely ability to pass through apertures of differing widths (in the motor imagery task) or to actually pass through them (in the action task). The action and motor imagery tasks used in the present study enabled us to quantify and statistically analyze what the clinician usually has to assess with "yes-no" questionnaire answers during a consultation.

Overall, our results showed that AN patients not only overestimated the critical aperture in the motor imagery task but also turned sideways for a wider aperture than controls when they had to really pass through the aperture. Firstly, the present overestimation in the motor imagery task confirms our previous results [15, 16] but was evidenced in a more ecological paradigm; here, patients were faced with a physical aperture rather than the projected (two-dimensional) doorway-like aperture used in our previous experiments.

Secondly, the similar passability overestimation for imagery and a real action task means that anorexic patients not only believe that they cannot pass through an aperture that is nevertheless wide enough for them but also behave as if this was the case. When the action was really performed, our results confirm the findings reported recently by Keizer et al. [17]. In the present experiment, both motor imagery and action impairments observed in the AN group provide evidence in favor of an overestimated body schema in this disorder. Moreover, the ratio did not change significantly during the real executed action (action task) and no motor optimization between the two series was evidenced. This result is not worthy for several reasons. In the action task, the patients might have been able to correct their motor imagery "error" when walking towards or through the aperture via integration of environmental cues [33, 35] into the motor program. A motor imagery "error" would consist of a participant turning her shoulders for an aperture that was easily wide enough to pass through without a postural change. After having performing a number of actual passages, the participant might also have been able to correct her "errors" by integrating feedback ("reality check") from previous trials [33]. In contrast, our present results suggest that the body schema alteration in AN patients was strong enough to resist correction by external cues and performance of the action.

Although our results argue strongly in favor of a body schema alteration in AN, the origin of this alteration is another question. In order to assess possible links between body schema disruption and the weight variation, we took account of the patient's weight before the onset of AN (as reported by the patients themselves), the weight one month and six months before the experiment and the weight at the date of the experiment. It must be noted that apart from the weight before the disease onset, the measurement during the course of the disease were made during normal medical care (i.e. during the weight recovery process). According to the "*allocentric lock*" hypothesis [36], anorexic patients could be trapped in an outdated body schema – perhaps because of rapid, massive weight loss. However, we did not have any information about body schema evaluation prior to the onset of AN. It is likely that the patient's body schema was already impaired. Moreover, the absence of a significant positive correlation between bodyweight before AN and either of the two passability ratios argues against the "*allocentric lock*" hypothesis. Nevertheless, the presence of certain study limitations means that we cannot totally reject this hypothesis. In particular, the strong emotional burden attached to the self-reported "weight before the disease" might be a major source of bias. This is already indeed the case in the general population [40]. Even though AN patients self-reported their weight very precisely (to within a gram, according to our data; Table 2), they may have overestimated this weight (probably unconsciously) in order to reduce the amount of weight they had to recover and/or because they had considered themselves to be too fat before the onset of AN.

By contrast, we found a significant, positive correlation between weight recovery (i.e. the current BMI less the minimum BMI) and the motor imagery ratio; the greater the weight recovery during the preceding six months, the higher (and thus more biased) the motor imagery ratio. This unexpected result prompted one of the present article's reviewers to suggest a new hypothesis. In anorexic patients, weight regain (with calorie fear and false beliefs about being "obese") is associated with limbic and paralimbic activation and has consequences on emotional arousal (i.e. the presence of obsessive and depressive symptoms) [41]. During the weight recovery phase, the AN inpatient group's depression and anxiety scores were pathological. One could imagine that this emotional arousal interferes with visual and somesthetic sensory inputs that underpin body perception and motor expression. This hypothesis should be testing in future research (particularly in neuro-imaging studies).

Our analysis did not enable us to identify the origin of the distorted body schema. Hence, links between weight variation and body schema distortions should be investigated further. For instance, longitudinal studies over 6, 12 or 18 months would probably provide useful information on this question. However, in view of the rapid weight loss and/or regain in AN, only a longitudinal study running from the pre-puberty period (before the onset of AN) to adulthood (after AN) could clearly identify whether the distortion in the body schema was longstanding or recently acquired.

Although the above results again confirmed the role of body schema in both action and motor imagery tasks in AN, the present study had several limitations. Firstly, the small sample size and the heterogeneity of the patient group (in terms of ethnic and geographic factors, and prior hospitalizations and treatments) may have reduced the power of our analysis. Secondly, we were not able to test influence of the duration of AN, since its range was too limited (only two participants had suffered from AN for more than 10 years). Thirdly, for technical reasons, the apertures were not randomly presented. This is especially important for patients with AN, since the latter display less cognitive flexibility than controls [42, 43]. Although we cannot completely rule out an effect of non-random presentation on performance in the motor action task, it is noteworthy that our present results for the motor imagery task, are very similar to those found previously with random sequences [15, 16]. Lastly, cautious behavior in the AN group (turning the shoulders or not expecting to pass through the aperture) might mirror the AN patients’ obsession with perfectionism [44, 45]. However, we checked for correlations between perfectionist traits (evaluated on the EDI-2's "perfectionism" subscale) and the two ratios of passability in the AN group. None achieved statistical significance (motor imagery ratio: ρ = -0.32, p > .20; action ratio: ρ = -0.26, p > .20). Even the non-significant trends in these correlations were negative: the higher the perfectionism score, the lower the ratios. Hence, it is difficult to infer that the higher ratios in the AN group were entirely due to perfectionist traits.

## Conclusions

In conclusion, a false feeling of being fat (a key symptom of AN) is not only confined to a conscious image of oneself but might affected the basic, sensorimotor representation of the body. This sensorimotor distortion might be modulated by the amount of weight recovery. The overall results of our experiment, even if they do not afford a new theoretical idea, reinforce the assumption of a body schema alteration in this psychiatric pathology. Our present findings may also open up new scientific perspectives. Future longitudinal studies will enable us to better understand (i) the origin of body schema distortion and (ii) the possible link between body schema distortion and the frequency of relapse (i.e. loss of weight after hospitalization, and maintenance of restraining behaviors).

Given that our present findings emphasize the key role of body schema in this psychiatric disorder, they also indicate the likely importance of evaluating and modulating the body schema in AN. In this perspective, therapies based on direct physical body experiences (physiotherapy, massage therapy, etc.) might be particularly valuable for countering body size overestimation in AN [46, 47]. In the same way, the use of virtual reality methods (in which body representation can be extensively manipulated [48]) represent key therapeutic perspectives for body schema rehabilitation in AN. We suggest that these virtual reality therapies should be based on body-scaled actions. By consciously confronting the patients with what they think they can (or cannot) do and what they can actually do (i.e. being able to pass through an appropriately wide aperture without turning their shoulders, for instance), it may be possible to improve their body representation. Indeed, the benefits of combining virtual reality treatment with cognitive behavioral therapies has been proven, with maintenance of these benefits after one year of follow-up [49].

## Abbreviations

AN: Anorexia Nervosa
BMI: Body Mass Index
DSM: Diagnostic and Statistical Manual of Mental Disorders
BSQ: Body Shape Questionnaire
EDI: Eating Disorder Inventory.
